# Real-life experience of tildrakizumab 200 mg in complex psoriatic patients: a case series

**DOI:** 10.3389/fmed.2026.1788690

**Published:** 2026-07-14

**Authors:** Giulia Odorici, Cinzia Buligan, Carolina Fantini, Lucia Mantovani, Giulia Rech, Lidia Sacchelli

**Affiliations:** 1 Section of Dermatology and Infectious Diseases, Department of Medical Sciences, University of Ferrara, Ferrara, Italy; 2 Institute of Dermatology, Azienda Sanitaria Universitaria Friuli Centrale (ASUFC), Udine, Italy; 3 Section of Dermatology, Department of Medicine and Surgery, University of Parma, Parma, Italy; 4 Division of Dermatology, Psoriasis Outpatient Service, APSS, Trento, Italy; 5 Dermatology Unit, IRCCS Azienda Ospedaliero-Universitaria di Bologna, Bologna, Italy; 6 Department of Medical and Surgical Sciences Alma Mater Studiorum, University of Bologna, Bologna, Italy

**Keywords:** biologics, comorbidities, difficult-to-treat, multi-failure, obese, overweight, psoriasis, tildrakizumab

## Abstract

**Background/Objectives:**

The management of moderate-to-severe plaque psoriasis may be particularly challenging in patients with a high disease burden and multiple comorbidities. This case series provides real-world evidence on the effectiveness of tildrakizumab 200 mg in patients with moderate-to-severe psoriasis characterized by clinical factors such as multiple comorbidities, high body weight, involvement of difficult-to-treat areas, and prior therapeutic failures.

**Methods:**

We present a series of six clinical cases describing baseline characteristics, treatment history and outcomes of patients with moderate-to-severe psoriasis treated with tildrakizumab 200 mg.

**Results:**

Although most of the patients included in this report showed multiple chronic or acute comorbidities (mainly cardiometabolic), high body weight, involvement of difficult-to-treat areas and failure to previous treatments (including biologics), tildrakizumab induced mostly complete remissions with most patients achieving PASI90 or PASI100 responses within 4–12 weeks; complete clearance (PASI100) was observed in four out of six patients. Treatment effectiveness was maintained over a follow-up of approximately 24 months in most cases, with a favorable safety profile.

**Conclusions:**

Tildrakizumab 200 mg demonstrated high effectiveness and sustained clinical responses in patients with moderate-to-severe psoriasis characterized by high disease burden and multiple comorbidities in a real-world setting.

## 1 Introduction

Psoriasis is a chronic inflammatory disease of the skin, estimated to affect approximately 125 million people worldwide, with highly variable prevalence evident between different regions (0.5%−8%) (1). In clinical practice, different factors such as the presence of comorbidities, the involvement of high impact areas, high body weight or obesity, as well as failure to prior therapies can negatively impact patient outcomes (2, 3). Multiple studies have reported the frequent association of psoriasis with different comorbidities, such as psoriatic arthritis, cardiovascular diseases (stroke, myocardial infarction, peripheral vascular disease), diabetes, chronic pulmonary disease, inflammatory

bowel disease, renal disease or psychological disorders (1, 4). In this context, the impact of systemic treatments, including biologics, needs to be carefully considered by dermatologists in accordance with the specific comorbidities affecting the patient, as highlighted by the EuroGuiDerm Guideline on the systemic treatment of psoriasis and the SIDeMaST (Italian Society of Dermatology and Sexually transmitted Diseases) adaptation of this guideline to the Italian healthcare context (5, 6). For example, it is well known that patients with psoriasis, especially those with moderate-to-severe psoriasis and concomitant metabolic conditions (such as obesity, non-alcoholic fatty liver disease/hepatic steatosis, and diabetes), may benefit from biological therapies (7). Consequently, real-life experience describing the effects of biologics in patients affected by specific comorbidities can provide useful information supporting physicians in managing these patient subpopulations.

Psoriasis typically affects the trunk, gluteal fold, and extensor surfaces of limbs. Nevertheless, the frequent involvement of high impact areas, such as the scalp, face, palmoplantar regions, intertriginous areas, nails, or genitals, can severely affect patient quality of life (8). A recent real-world study showed that only a limited number of patients with the involvement of high impact areas are treated with biologics (9), although biologic agents are known to be highly effective on psoriasis localized in these special regions (10–12).

In recent years, multiple biologic agents targeting cytokines with key roles in the pathophysiology of psoriasis (in particular interleukin IL-17 and IL-23) have been developed and approved for the treatment of the disease. Nevertheless, despite the generally higher treatment persistence observed with biologic therapies compared with conventional systemic treatments, a subset of patients may experience primary non-response, secondary loss of efficacy, or tolerability issues over time, which can lead to treatment discontinuation and switching to alternative biologic agents (12). Interestingly, a recent real-world study comparing the activity of different biologics demonstrated that anti-IL-23 agents were significantly associated with the lowest switching risk over a treatment period of 24 months (13), underlying the good performance of this class of biologics in real-life. Anti-IL-23 agents are among the most recent class of biologics approved for moderate-to-severe psoriasis, able to impact the Th17 immune pathway reducing the production of associated pro-inflammatory cytokines (IL-17A-F, IL-22, IL-21, TNF-alfa) (1, 14). Among anti-IL-23 agents, tildrakizumab is a humanized monoclonal antibody specifically inhibiting IL-23p19 interaction with its receptor, without interfering with IL-12 (15). Tildrakizumab was approved by the European Medicines Agency (EMA) in 2018 for the treatment of adults with moderate-to-severe plaque psoriasis eligible to systemic therapy (16). Interestingly, alternatively to the standard dose of 100 mg, tildrakizumab distinctive dosage flexibility allows to administer a dose of 200 mg at the physician's discretion, potentially improving clinical outcomes for patients with a bodyweight >90 kg or those with a high disease burden (2).

Despite the availability of several biologic therapies, evidence on the management of patients with multiple comorbidities in real-world settings remains limited. The aim of this study is to describe the effectiveness and safety of tildrakizumab 200 mg in a series of patients with moderate-to-severe psoriasis presenting features associated with “clinical complexity”, intended as a clinical profile characterized by a high burden of disease, including the involvement of difficult-to-treat areas (such as the scalp, face, palmoplantar regions, intertriginous areas, nails, or genitals), the presence of multiple comorbidities (both well-recognized psoriasis-associated conditions, such as cardiometabolic disorders, non-alcoholic fatty liver disease/hepatic steatosis, and psoriatic arthritis, and less commonly described conditions, such as previous or current malignancy, indolent systemic mastocytosis, previous severe infections, or acute respiratory failure), high body weight or obesity, and failure to prior systemic therapies, including biologic therapies. This operational definition reflects the reality of routine clinical practice, where treatment selection must not only ensure efficacy against psoriasis but also guarantee an adequate safety and tolerability profile with respect to the patient's concomitant clinical conditions.

## 2 Materials and methods

In the present article we describe six clinical cases of patients with moderate-to-severe psoriasis selected from routine clinical practice at tertiary dermatology centers in Italy between 2022 and 2024. Patients were selected based on the presence of multiple acute or chronic comorbidities, involvement of high impact areas and previous failure of topical therapies, conventional systemic therapies as well as biologics (Table 1). These cases were not intended to represent a consecutive series, but rather illustrative examples of patients requiring individualized therapeutic strategies. All the patients were ultimately treated with tildrakizumab 200 mg administered by subcutaneous injection at weeks 0, and 4 and then every 12 weeks, based on clinical judgement taking into account disease severity, patient characteristics and limited therapeutic alternatives. Tildrakizumab effectiveness was evaluated by recording the Psoriasis Area and Severity Index (PASI) and Dermatology Life Quality Index (DLQI) at different timepoints as indicated in Table 1.

**Table 1:** Patient characteristics and outcomes.

Patients characteristics	Case 1		Case 2		Case 3		Case 4		Case 5		Case 6	
Age	75		78		40		56		53		43	
Sex	M		M		F		M		M		M	
Smoker	Yes		No		Yes		Yes		Just stopped		No	
Pso duration	50 year		30 year		10 year		26 year		40 year		20 year	
Weight	70 kg		140 kg		90 kg		97 kg		115 kg		95 kg	
BMI	24.2		40		35		30		41		26.4	
Previous therapies	8-MOP PUVA, systemic CS and cetirizine, apremilast		Topical CS, systemic CS, acitretin, NB-UVB, MTX, DMF		NB-UVB, bath treatments, CyA		NB-UVB, CyA		PUVA, MTX		Topical CS, MTX	
Biologics	No		Etanercept 1 year		Ixekizumab 6 year		Adalimumab 6 year		Ixekizumab 8 month		No	
					Brodalumab 6 month		Secukinumab 3 year		Ixekizumab + MTX 5 month			
Initiation of tildrakizumab therapy (duration)	2023		2021		2023		2023		2022 + MTX 10 mg (12 month)		2023	
Tildrakizumab dose	200 mg		100 mg → 200 mg		200 mg		200 mg		200 mg		200 mg	
Psoriasis manifestation (special sites)	Sub-erythrodermic		Scalp, nails, genitalia				Scalp, face		Scalp, nails		Scalp, nails	
Comorbidities	Hypertension, PAOD, COPD, Coronary ischemic heart disease with severe left ventricular dysfunction, urothelial neoplasm, MRSA sepsis		Metabolic syndrome, hypothyroidism, microcytic anemia TT, myelodysplastic syndrome, gastric ulcer hepatic steatosis, chronic kidney failure, PAOD paraseptal pulmonary emphysema, osteonecrosis of the femoral head and prosthesis (dehiscence of the surgical wound, infection from *K. pneumoniae* and *P. aeruginosa*)		Obesity, gastric banding, Hashimoto thyroiditis, psoriatic arthritis, indolent systemic mastocytosis with dermatological involvement		Hyperlipidemia		Metabolic syndrome, obesity, OSA syndrome, COPD, polycythemia DM type II, hypertension, acute respiratory failure, and chronic heart failure due to dilated myocardiopathy		None	
Peculiarity	Sub-erythrodermic psoriasis, multi-failure, urothelial neoplasm, MRSA sepsis		high impact areas, obese/overweight, multi-failure (including biologics), >65 year, osteonecrosis of the femoral head (followed by prosthetic replacement and infection), remission after tildrakizumab withdrawal		Obese/overweight, multi-failure (including biologics), mastocytosis		Obese, high impact areas, multi-failure (including biologics)		Acute respiratory failure and chronic heart failure		High impact areas, overweight	
Outcome	Case 1		Case 2		Case 3		Case 4		Case 5		Case 6	
	Timepoint	Score	Timepoint	Score	Timepoint	Score	Timepoint	Score	Timepoint	Score	Timepoint	Score
Baseline PASI		Sub-erythrodermic		24		12		20		50		6.8
T1 PASI	Week 4	10	Week 4	12	Week 4	0	Week 12	0	Week 12	7	Week 4	1
T2 PASI	Week 8	0	Week 8	6							Week 22	0
T3 PASI			Week 12 (200 mg)	0								
Baseline DLQI		21		NA		14		10		>10		NA
T1 DLQI	Week 4	7	Week 4	NA	Week 4	0	Week 12	0	Week 12	NA	Week 4	NA
T2 DLQI	Week 8	0	Week 8	NA							Week 22	NA
T3 DLQI			Week 12 (200 mg)	NA								

8-methoxypsoralen (8-MOP); BMI, Body Mass Index; COPD, chronic obstructive pulmonary disease; CS, corticosteroid(s); CyA, cyclosporine; DLQI, Dermatology Life Quality Index; DM, diabetes mellitus; DMF, dimethyl fumarate; high impact, difficult-to-treat; F, female; m, month(s); M, male; MRSA, methicillin-resistant Staphylococcus aureus; MTX, methotrexate; NA, data not available; NB-UVB, narrow-band ultraviolet-B; OSA, obstructive sleep apnea; PAOD, peripheral arterial occlusive disease; PASI, Psoriasis Area and Severity Index; Pso, psoriasis; PUVA, photochemotherapy; TT, thalassemia trait; VAS, visual analog scale; y, year(s).

## 3 Results

### 3.1 Case series

#### 3.1.1 Case 1

**Figure 1:**
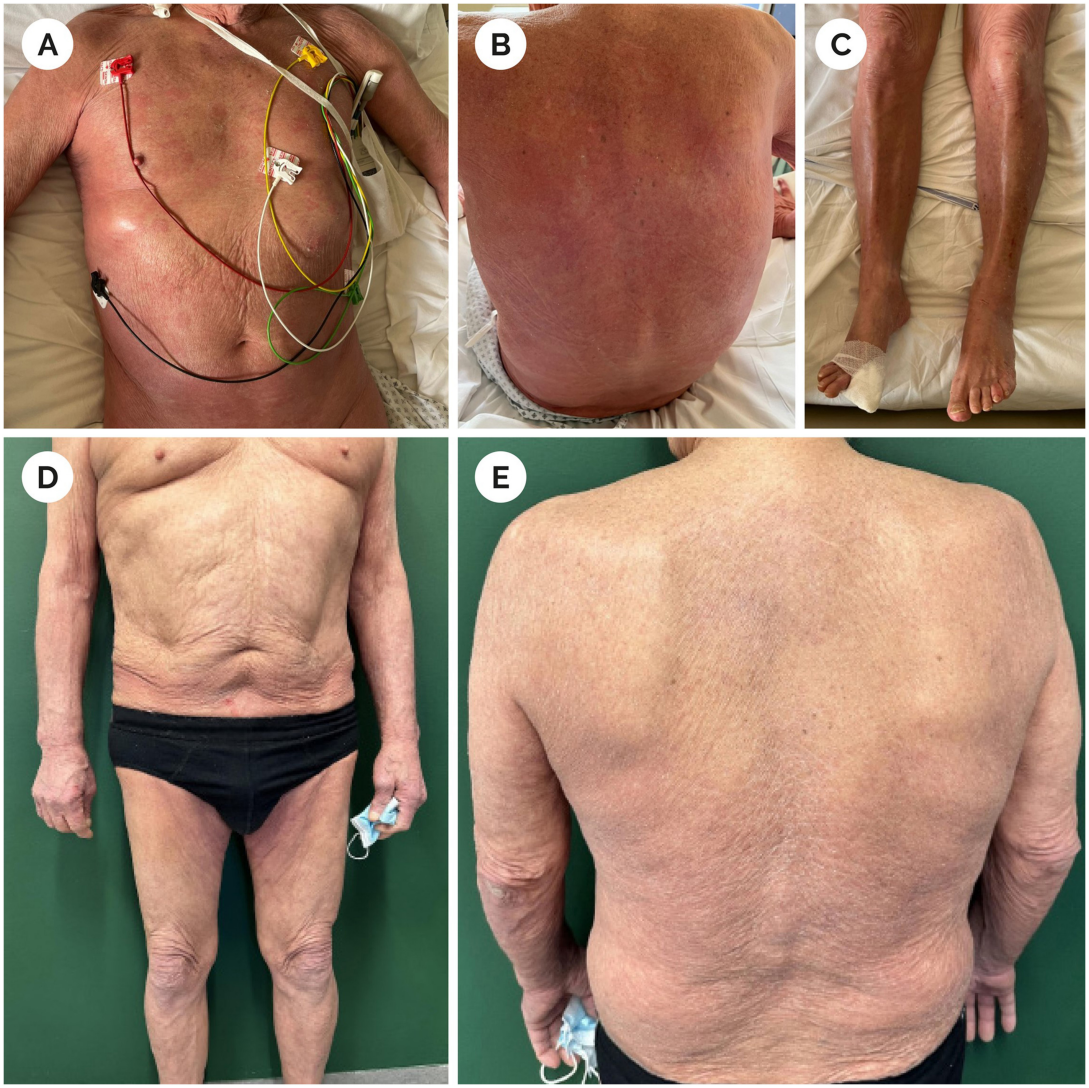
Clinical benefits following tildrakizumab 200 mg therapy in a patient with sub-erythrodermic psoriasis and multiple comorbidities. **(A–C)** Initial manifestations of sub-erythrodermic psoriasis. **(D, E)** Complete remission reached after 8 weeks of treatment with tildrakizumab 200 mg.

A 75-year-old Caucasian man with a 50-year history of plaque psoriasis was examined in our clinic for the presence of sub-erythrodermic psoriasis. The patient had a body weight of 70 kg [body mass index (BMI) = 24.2], and multiple comorbidities including hypertension, peripheral arterial occlusive disease (PAOD), chronic obstructive pulmonary disease (COPD), high grade papillary urothelial carcinoma, ischemic heart disease with severe left ventricular dysfunction, as well as methicillin-resistant *Staphylococcus aureus* (MRSA) sepsis (Table 1). The patient was previously treated for psoriasis with oral corticosteroids, oral 8-methoxypsoralen (8-MOP) photochemotherapy (PUVA), and apremilast (3 months) resulting in limited efficacy (sub-erythrodermic at baseline and DLQI = 21). Subsequently, a therapy based on tildrakizumab 200 mg was initiated, resulting in the achievement of a complete remission after 8 weeks (PASI100 and DLQI = 0; Figure 1).

#### 3.1.2 Case 2

**Figure 2:**
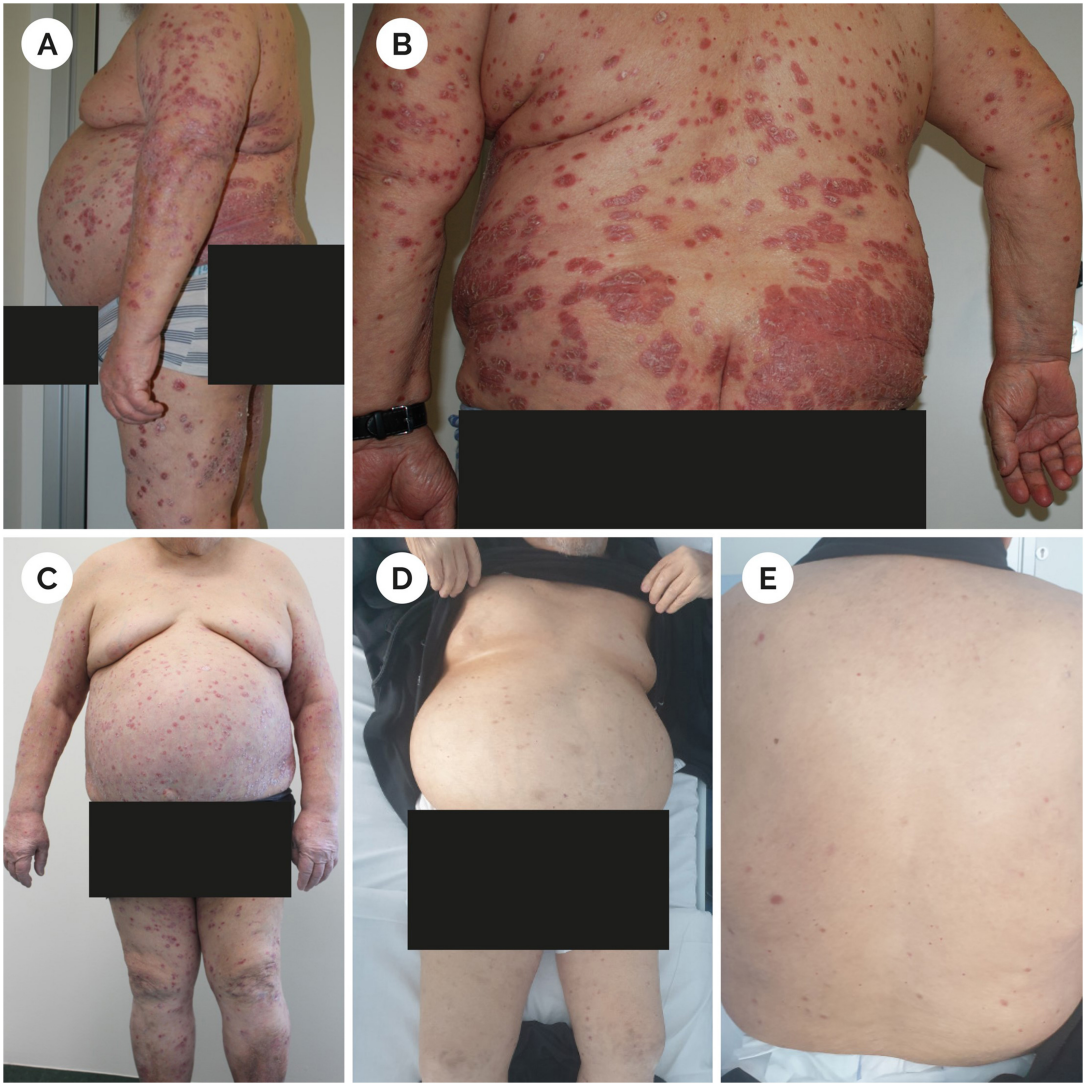
Clinical benefits after treatment with tildrakizumab 100 mg and 200 mg in an obese and multi-failure patient with plaque psoriasis and multiple comorbidities**. (A, B)** Presentation of the patient before initiating tildrakizumab therapy. **(C)** Clinical benefits after 4 weeks of therapy with tildrakizumab 100 mg. **(D, E)** Complete remission reached after 12-week treatment with tildrakizumab 200 mg.

A 78-year-old Caucasian man with a 30-year history of psoriasis with involvement of high impact areas (scalp, nails, and genitalia) was examined in our clinic. The patient's body weight was 100 kg at the first visit, reaching 140 kg (BMI >30) during treatment. Multiple comorbidities were reported, including metabolic syndrome (diabetes, grade III obesity, dyslipidemia, and hypertension), hypothyroidism, gastric ulcer, hepatic steatosis, microcytic anemia (thalassemia, Hb 9 g/dl, MCV 73 fl) and myelodysplastic syndrome with unilineage dysplasia and normal karyotype treated with erythropoietin-based therapy (Table 1). The patient was previously exposed to multiple therapies, including topical drugs, narrow-band ultraviolet-B (NB-UVB; followed by sub-erythrodermic psoriasis manifestation), conventional systemic drugs and etanercept, ultimately resulting in PASI = 24 (basal scores). Subsequently, tildrakizumab 100 mg was administered, resulting beneficial already at week 4 (PASI50) and leading at week 8 to further improvements (PASI75; Figure 2). The patient experienced osteonecrosis of the femoral head requiring a replacement with a prosthetic head, followed by wound dehiscence and infection with *K. Pneumoniae* e *P. Aeruginosa*, successfully treated with negative pressure wound therapy. Considering the augmented body weight and high BMI of the patient, tildrakizumab dose was increased (200 mg) resulting in complete remission (PASI100) after 12 weeks (Figure 2).

#### 3.1.3 Case 3

A 40-year-old Caucasian woman with 10-year history of psoriasis was examined in our clinic. The patient's characteristics included a body weight of 90 kg (BMI = 35), previous gastric band surgery, Hashimoto's thyroiditis, and indolent systemic mastocytosis with dermatological involvement (tryptase = 20 mcg/L, *D816V* mutation, and mast cells CD2+ and CD25+) treated with antihistamines (Table 1). The patient was previously treated with NB-UVB, bath treatments, cyclosporine (CyA; discontinued for adverse events), as well as with anti-IL-17 agents. In particular, she received 6-year treatment with ixekizumab, showing an initial response followed by a loss of efficacy, and brodalumab, showing a loss of efficacy after treatment for 6 months. Considering the patient's therapeutic history and characteristics, treatment with tildrakizumab 200 mg was initiated (basal PASI = 12, DLQI = 14 and itch VAS = 6). Remarkably, complete remission (i.e., PASI100, DLQI = 0 and itch VAS = 0) was achieved after only 4 weeks of treatment.

#### 3.1.4 Case 4

**Figure 3:**
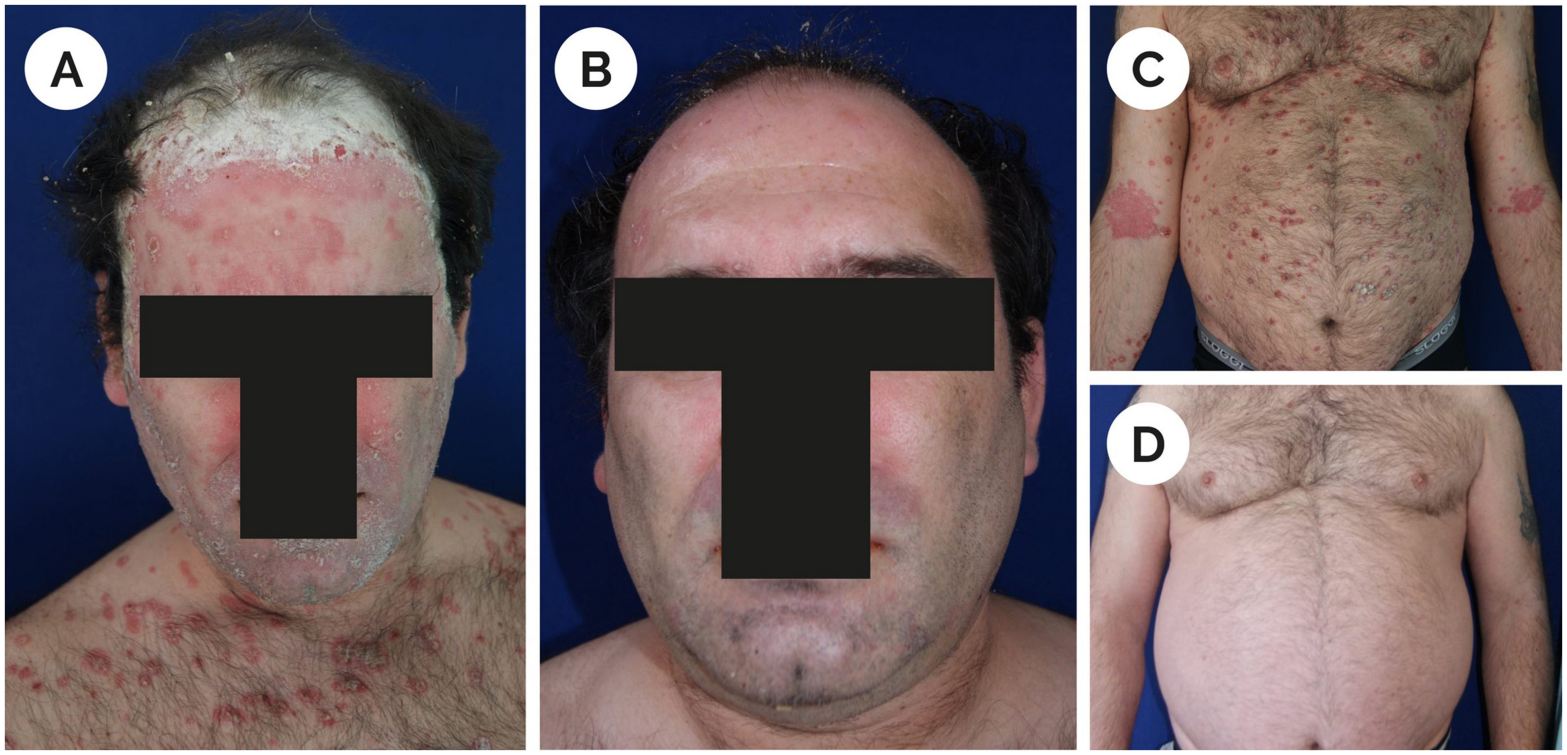
Complete remission of a patient with plaque psoriasis localized to the scalp, and the trunk after treatment with tildrakizumab 200 mg. **(A, C)** Psoriasis manifestation before initiating therapy with tildrakizumab 200 mg. **(B, D)** Complete remission after 12 weeks of treatment.

A 56-year-old Caucasian man with a 26-year history of psoriasis and psoriasis involving the scalp and the face along with typical areas was followed in our clinic. The patient's body weight was 97 kg (BMI = 30), and hypercholesterolemia was the only relevant comorbidity recorded (Table 1). The patient had previous received long-term treatments with NB-UVB and CyA, followed by therapy with adalimumab, which had initially provided good clinical control of the disease. Nevertheless, the switch to a biosimilar agent led to a loss of efficacy. Subsequently, secukinumab therapy (anti-IL-17) was administered for 3 years, but its efficacy diminished over time, resulting at the end of treatment in PASI = 20 and DLQI = 10 (Figure 3). Considering the patient characteristics (high BMI and multi-failure), tildrakizumab 200 mg was administered leading, after 12 weeks of treatment, to a complete remission of the disease (PASI100 and DLQI = 0; Figure 3).

#### 3.1.5 Case 5

**Figure 4:**
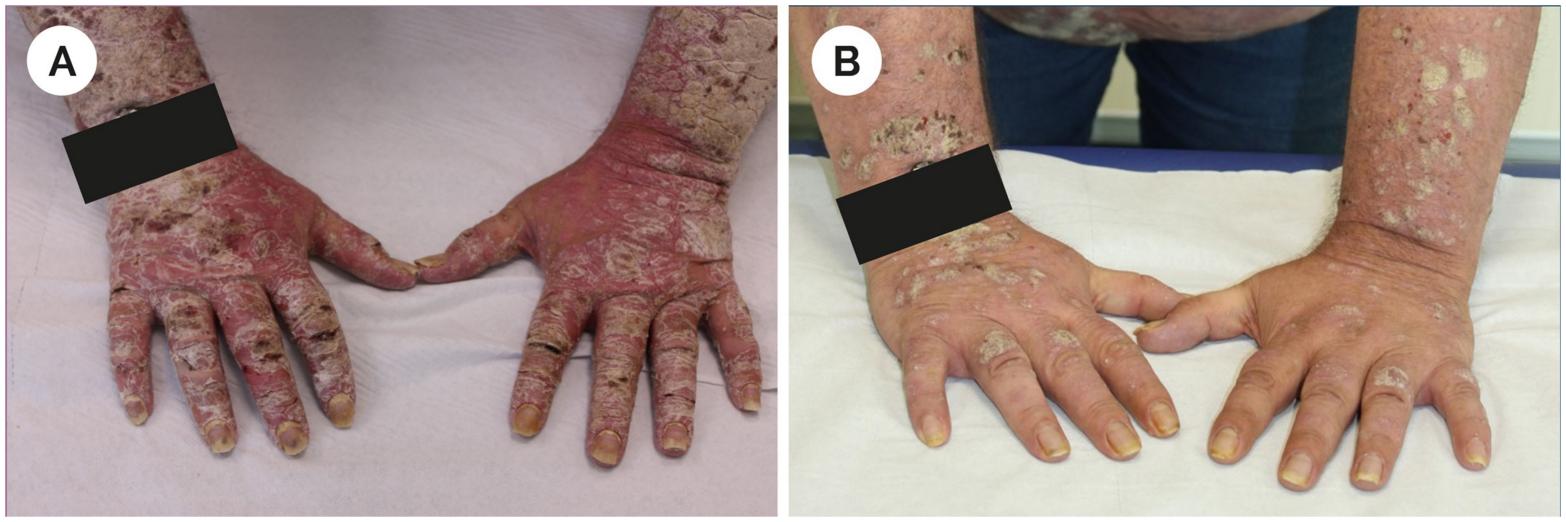
Clinical benefits following tildrakizumab 200 mg therapy in a patient with severe respiratory failure. (**A)** Hands involvement before tildrakizumab treatment. **(B)** Relevant benefits reached after 4 weeks of treatment with tildrakizumab 200 mg.

A 53-year-old Caucasian man with plaque psoriasis also involving high impact areas (scalp and nails) and 40-year history of the disease was examined in our clinic. The patient had a body weight of 115 kg (BMI = 41), and multiple comorbidities, including metabolic syndrome (diabetes mellitus type II, obesity, and hypertension), obstructive sleep apnea, COPD, and polycythemia. Moreover, the patient had chronic heart failure due to dilated myocardiopathy and experienced an acute respiratory failure. Previously conventional treatments for psoriasis (PUVA and MTX) were administered, followed by ixekizumab for 8 months plus methotrexate during the last 5 months. Despite the biological therapy and the additional MTX, insufficient disease control was evident. Considering the high burden of the disease, tildrakizumab 200 mg was administered, leading to significant clinical benefits [from basal PASI = 50 and DLQI>10 to residual PASI = 7 (PASI90 response)] after 12 weeks of treatment (Figure 4).

#### 3.1.6 Case 6

**Figure 5:**
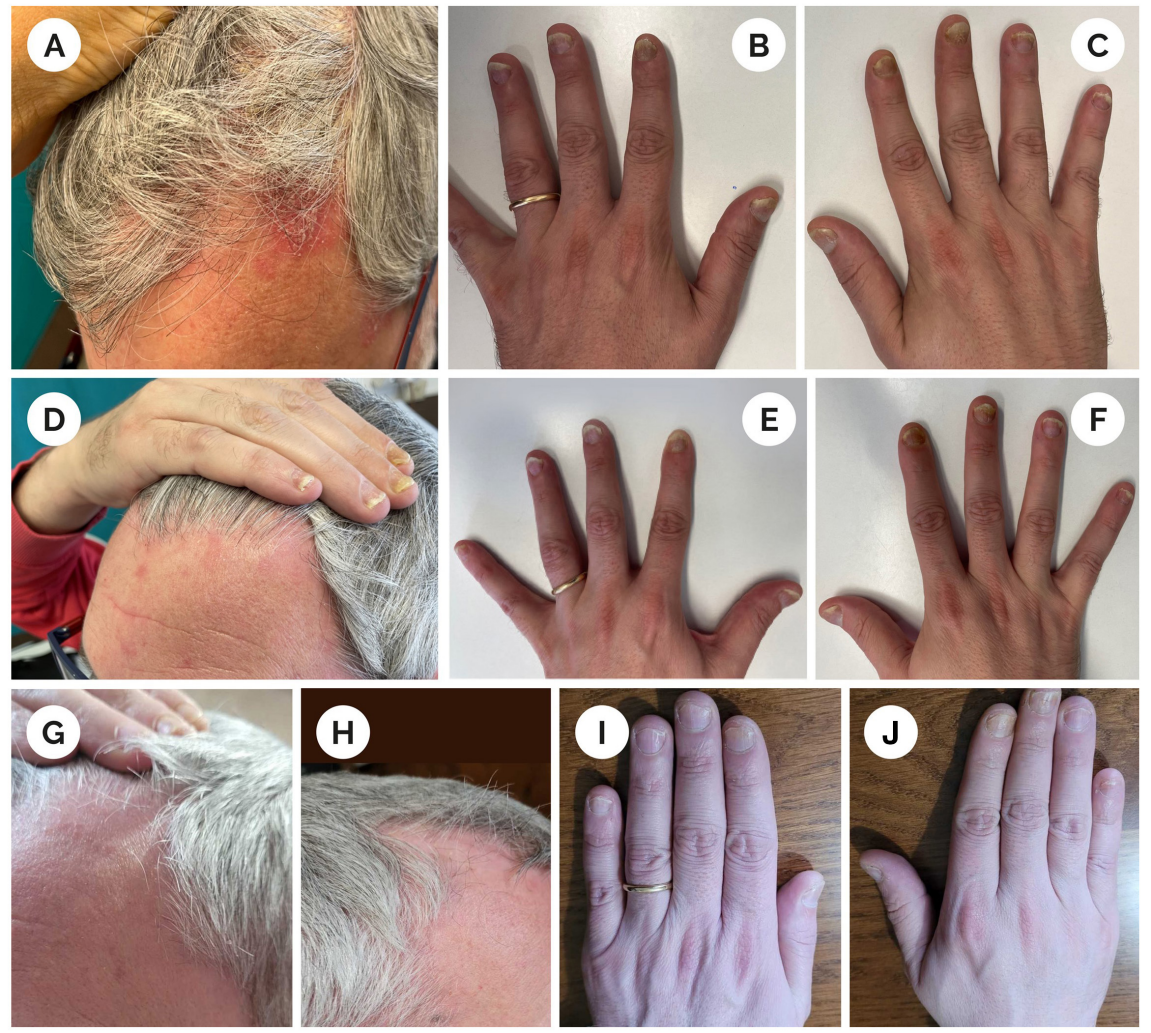
Clinical benefits following tildrakizumab 200 mg therapy in a patient with plaque psoriasis located on the scalp and nails. **(A–C)** Initial presentation of patient's scalp and nails. **(D–F)** Clinical benefits after 4 weeks of therapy with tildrakizumab 200 mg. **(G–J)** Complete remission reached after the 22-week treatment with tildrakizumab 200 mg.

A 43-year-old Caucasian man with psoriatic onychopathy and plaque psoriasis localized to the scalp was examined in our clinic. The patient had a 20-year history of psoriasis, body weight of 95 Kg (BMI = 26.4), and absence of relevant comorbidities (Table 1). The patient was previously treated with topical corticosteroids and methotrexate, discontinued after 6 weeks for nausea and asthenia. Subsequently, tildrakizumab 200 mg was selected on the basis of the patient's characteristics (body weight and involvement of high impact areas) and administered as previously described (see Materials and Methods). The patient was initially characterized by basal PASI = 6.8 and basal modified Nail Psoriasis Severity Index (mNAPSI) = 23 (Figure 5), and reached PASI90 and mNAPSI = 19 at week 4 (Figure 5). Finally, the patient achieved a complete remission after 22 weeks of treatment with tildrakizumab (PASI100 and mNAPSI = 2.3; Figure 5).

#### 3.1.7 Follow-up and safety observations

All six patients achieved a clinically meaningful improvement following initiation of tildrakizumab 200 mg, with rapid reduction in disease severity and improvement in quality of life parameters, as detailed in Table 1. Follow-up was heterogeneous across cases, reflecting real-world clinical practice; however, updated follow-up data were available for all patients and allowed exploratory assessment of treatment durability, persistence, and safety.

For Cases 1 and 6, at approximately 2 years of continuous treatment, tildrakizumab therapy is ongoing with sustained clinical benefit and without any reported safety concerns or treatment discontinuations.

In Case 2, tildrakizumab was discontinued due to an episode of sepsis considered unrelated to the study treatment. Notably, after treatment discontinuation, the patient maintained complete skin clearance (PASI 0) for approximately 2 years in the absence of any systemic psoriasis therapy. The patient subsequently died due to natural causes not related to psoriasis or its treatment.

In Case 3, at approximately 2 years from treatment initiation, the patient remains on tildrakizumab with sustained clinical response and no reported safety concerns. Treatment persistence was maintained throughout the follow-up period.

In Case 4, follow-up evaluation performed at about 2 years after treatment initiation confirmed ongoing disease control with minimal residual psoriasis (PASI 2), limited to small plaques on the pretibial area and scalp. No treatment-related adverse events were reported during this period.

In Case 5, the patient is currently continuing tildrakizumab therapy and remains clinically stable, achieving PASI 90. Despite experiencing two additional hospitalizations for respiratory insufficiency due to underlying comorbidities, treatment was not interrupted, and no drug-related safety issues were identified.

Overall, across the six reported cases, tildrakizumab 200 mg demonstrated sustained effectiveness and a favorable safety profile in a cohort of patients with moderate-to-severe psoriasis characterized by significant clinical complexity. Although long-term relapse rates were not formally assessed, available follow-up data support treatment persistence and durability of response in routine clinical practice.

## 4 Discussion

In the present case series, we describe patients affected by moderate-to-severe psoriasis presenting with a broad spectrum of comorbid conditions. Some of these, including cardiometabolic disorders, obesity, cardiovascular disease, and chronic pulmonary conditions, represent a well-recognized and typical clinical profile of patients with psoriasis, as consistently reported in epidemiological and real-world studies.

Conversely, other conditions observed in our cohort, such as solid tumors, indolent systemic mastocytosis with cutaneous involvement, previous severe infections, or acute respiratory failure, are less commonly described in association with psoriasis and are not considered typical comorbidities of the disease. These conditions were reported to highlight the clinical complexity of patients managed in real-life settings and to illustrate challenging therapeutic scenarios in which treatment selection may be particularly difficult. Regarding the impact of comorbidities on the management of patients with psoriasis, the selection of the most suitable biologic agent on the basis of concomitant conditions is a critical determinant for improving patient benefits (17). In this context, the presence of a current or previous malignancy represents one of the most challenging clinical scenarios in the treatment of moderate-to-severe psoriasis. Although patients with a history of cancer have traditionally been excluded from randomized clinical trials, an increasing body of real-world evidence supports the cautious use of biologic therapies in selected cases. Large observational and multicenter studies have reported low rates of cancer progression, recurrence, or new malignancy development in patients treated with biologics, including anti–IL-23 agents such as tildrakizumab, with safety profiles comparable to conventional systemic therapies. (17–19). Beyond oncological conditions, cardiometabolic conditions such as obesity, metabolic syndrome, cardiovascular diseases, and diabetes mellitus, are typically associated with psoriasis, sharing common pathogenic mechanisms involving genetic factors, insulin resistance, metabolism of lipids as well as inflammatory pathways (20). In particular, growing evidence highlights that the IL-23/Th17 immune pathway is also involved in several psoriasis-associated comorbidities, such as myocardial damage and stroke, diabetes, obesity, and non-alcoholic fatty liver disease. Consequently, biologics targeting this pathway could ideally benefit both psoriasis as well as associated comorbidities (14). Focusing specifically on tildrakizumab therapy, the prevalence of dyslipidemia, hypertension, cardiovascular diseases, diabetes or obesity have been frequently reported in patients recruited in pivotal clinical trials as well as in real world studies testing this biologic agent (21–23). *Post-hoc* analyses of clinical studies reported that efficacy and safety of tildrakizumab were generally similar in patients with or without metabolic syndrome, and cardiometabolic risk factors did not increase following tildrakizumab treatment, confirming its favorable safety profile in these patients (24, 25). More recently, Narcisi and co-workers, in a sub-analysis of a large Italian real-world study, highlighted that tildrakizumab was significantly more effective in patients with cardiovascular or metabolic comorbidities, further supporting its use in this patient subpopulation (22).

The use of tildrakizumab 200 mg has been increasingly explored registering potential additional benefits in patients ≥90 kg or with high burden of the disease (including the involvement of high impact areas, high PASI score and/or failure of previous therapies including biologics) (2). Regarding overweight patients, a pooled analysis over 5 years of the pivotal phase three clinical studies (reSURFACE 1 and 2) highlighted a favorable trend in term of PASI and DLQI responses of tildrakizumab 200 mg compared with tildrakizumab 100 mg in patients with a body weight ≥90 kg (26). More recently, a real-world comparative study confirmed and strengthened these observations, providing evidence of a significantly higher response of tildrakizumab 200 mg in terms of PASI90 and PASI100, especially at week 16, 28 and 52, in patients ≥90 kg (27).

Considering the therapeutic effectiveness of tildrakizumab in complex patients with psoriasis with involvement of high impact, real-word evidence confirmed the favorable results obtained in our case reports using the 200 mg dose. The study performed by Valenti and coworkers highlighted significantly higher PASI responses in patients with involvement of these areas treated with tildrakizumab 200 mg compared with tildrakizumab 100 mg at week 16 and 52 (27). Moreover, the real-world study conducted by Dattola and co-workers showed that tildrakizumab 200 mg was highly effective on psoriasis affecting genitalia and nails (28). Additional real-world studies further support the use of tildrakizumab in patients with severe pre-existing conditions and complex clinical profiles. In particular, Bardazzi and co-workers, reported tildrakizumab effectiveness in patients characterized by the involvement of high impact areas, BMI ≥30, psoriatic arthritis or ≥3 comorbidities as well as prior failure of biologic treatment (21).

It should be acknowledged that other biologic classes, including anti-TNF, anti-IL-17, and other anti-IL-23 agents, have also demonstrated high efficacy in patients with moderate-to-severe psoriasis and relevant comorbidities. However, in clinically complex patients, treatment selection is often driven not only by efficacy but also by long-term safety, tolerability, and comorbidity-specific considerations. In this context, IL-23 inhibitors have emerged as a particularly suitable option in patients with cardiometabolic disease, previous malignancy, or history of severe infections (6, 12).

Notably, the Living EuroGuiDerm Guideline (6) for the systemic treatment of psoriasis vulgaris specifically addresses the management of patients with relevant comorbidities and special clinical situations, emphasizing the importance of individualized therapeutic decisions. In this framework, the selection of biologic agents with a favorable safety profile, such as IL-23 inhibitors, is supported in patients with cardiometabolic disease, previous malignancy, or other complex conditions. Within this class, tildrakizumab further enables treatment personalization through its two approved dosing regimens, offering clinicians additional flexibility to tailor the therapeutic approach according to patient-specific characteristics, disease severity, and comorbidity profile.

Despite these encouraging findings, some limitations should be acknowledged. First, the small number of patients limits the generalizability of the findings and precludes any formal assessment of efficacy, durability of response, or long-term safety. In addition, the descriptive and non-consecutive case selection may introduce selection bias, while follow-up duration was heterogeneous among patients. Furthermore, pharmacoeconomic considerations were not formally assessed in the present study and no cost–effectiveness analysis was performed. However, it can be hypothesized that the use of tildrakizumab in patients with high disease burden may have relevant implications from a healthcare resource perspective. In particular, the favorable safety and tolerability profile reported in clinical trials and real-world studies may potentially translate into a reduced risk of treatment-related complications, a lower need for intensive laboratory monitoring, and simplified management compared with other systemic therapies. These aspects could contribute to a more efficient use of healthcare resources, including fewer visits, reduced need for laboratory assessments, and lower overall management burden. However, dedicated pharmacoeconomic studies are required to confirm these assumptions. Nevertheless, the study provides clinically meaningful real-world evidence on the use of tildrakizumab 200 mg in a population of patients with particularly complex clinical profiles who are often underrepresented in randomized clinical trials.

## 5 Conclusions

In the present report, we highlighted the rapid effectiveness of tildrakizumab 200 mg in six patients with moderate to severe plaque psoriasis frequently characterized by involvement of high impact areas, body weight ≥90 kg or BMI ≥30, and prior failure to multiple lines of treatment with conventional therapies or biologics. Although the presence of multiple chronic or acute comorbidities was mostly reported in these patients, tildrakizumab demonstrated high effectiveness as well as a favorable safety profile.

## Data Availability

The original contributions presented in the study are included in the article/supplementary material, further inquiries can be directed to the corresponding author.
